# Reliability and validity of the Vietnamese version of the Hamilton D-17 scale

**DOI:** 10.3389/fpsyt.2023.1089473

**Published:** 2023-04-06

**Authors:** Phuong Le-Nguyen-Thuy, Trang Nguyen-Dao-Uyen, Anh Tran-Nguyen-Quynh, Truc Thanh Thai, Linh Ngo-Tich, Dung Do-Van, Sy Duong-Quy

**Affiliations:** ^1^Division of Psychiatry, Department of Medicine, University of Medicine and Pharmacy at Ho Chi Minh City, Ho Chi Minh City, Vietnam; ^2^Department of Family Medicine, University of Medicine and Pharmacy at Ho Chi Minh City, Ho Chi Minh City, Vietnam; ^3^Faculty of Public Health, University of Medicine and Pharmacy at Ho Chi Minh City, Ho Chi Minh City, Vietnam; ^4^Bio-Medical Research Center, Lam Dong Medical College, Da Lat, Vietnam; ^5^Hershey Medical Center, Penn State Medical College, Hershey, PA, United States; ^6^Outpatient Department, Pham Ngoc Thach Medical University, Ho Chi Minh City, Vietnam; ^7^Department of Respiratory Functional Exploration, University Medical Center, University of Medicine and Pharmacy at Ho Chi Minh City, Ho Chi Minh City, Vietnam

**Keywords:** Hamilton D-17 scale, Vietnamese version, reliability, validity, depression

## Abstract

**Background:**

While depression is a common mental disorder, the diagnosis of this condition is still challenging. Thus, there is a need to have a validated tool to help evaluate symptoms of depression. This study aimed to evaluate the reliability and validity of the Vietnamese version of the Hamilton D-17 scale.

**Methods:**

A cross-sectional, descriptive, and validation study was conducted on 183 patients including 139 depressed and 44 non-depressed patients at the University Medical Center of Medicine and Pharmacy University at Ho Chi Minh City. Internal reliability and inter-rater reliability was measured using Cronbach's alpha and intraclass correlation coefficients (ICC). Confirmatory factor analysis (CFA) was used to evaluate construct validity. The Patient Health Questionnaire (PHQ9) was used to measure concurrent validity of the Hamilton D-17. Area under the ROC curve was used to measure criterion validity.

**Results:**

Both Cronbach alpha coefficient and ICC were at good level at alpha = 0.83 and ICC = 0.83. CFA with a second-order model consisting of four factors fitted the data at good to excellent level. The SRMR (Standardized Root Mean Squared Residual) was 0.066, RMSEA (Root Mean Square Error of Approximation) (90% CI) was 0.053 (0.036–0.069), CFI (comparative fit index) was 0.93, TLI (Tucker Lewis index) was 0.92. The Hamilton D-17 and the PHQ-9 had a correlation coefficient of *r* = 0.77 (*p* < 0.001). The Hamilton D-17 had a very high level of criterion validity with AUC of 0.93 (0.88–0.98).

**Conclusion:**

The Vietnamese version of the Hamilton D-17 scale has a high level of validity and reliability. The scale should be used to assess symptoms of depression among Vietnamese patients.

## Introduction

Depression is a common mental disorder, with an estimate of 300 million people, equivalent to 4.4% of the global population living with this condition (1). Unlike other diseases, such as diabetes, hypertension, hypothyroidism, and hyperthyroidism, depressive disorder has no subclinical tests to assess its severity and to monitor the effectiveness of the treatment. Thus, identification of depressive disorder is based primarily on clinical evaluation or scales. To date, there are many depression assessment scales currently used in clinical practice, such as the Beck Depression Inventory (BDI), the Patient Health Questionnaire-9 (PHQ-9), the Center for Epidemiological Studies Depression Scale (CES-D), the Zung Self-Rating Depression Scale (SDS) and the Hamilton D-17 (2–5). However, the BDI, PHQ-9, CES-D, and SDS are based on the patient's subjective perceptions (6). In contrast, the Hamilton D-17 scale assesses the severity of depression based on clinician's evaluation (5).

The Hamilton D-17 is a common scale and has been considered as a standard scale for assessing the severity of depression. Additionally, the Hamilton D-17 has also been used to measure the effectiveness of depression treatment in many studies (7–9). The Hamilton D-17 has been translated into many languages such as Turkish, Chinese, and Spanish. A high level of reliability and validity of the scale in different languages has been reported in previous validation studies (10–13).

In Vietnam, it is estimated that the prevalence of depression in general population is about 4.0% (14). In clinical practice, the majority of Vietnamese clinicians evaluate symptoms of depression based primarily on their experiences and patients'clinical symptoms. Despite the presence of DSM-IV and recently DSM-5, diagnosis of depression remains a challenge. Thus, disagreement in diagnosis of depression is common in the country. Moreover, without a standardized and validated scale such as the Hamilton D-17, it is also hard to evaluate the effectiveness of depression treatment. Several studies have been conducted in Vietnam using the Hamilton D-17 to evaluate depression.^1^ However, the psychometric properties of the scale have not been reported. The lack of such validation prevents clinicians from using the scale in their routine diagnosis and treatment.

Therefore, this study was conducted to investigate psychometric properties of the Vietnamese version of the Hamilton D-17 scale. These properties included internal reliability, inter-rater reliability, construct validity, concurrent validity and criterion validity.

## Methods

### Translation of the Hamilton D-17

**Figure 1:**
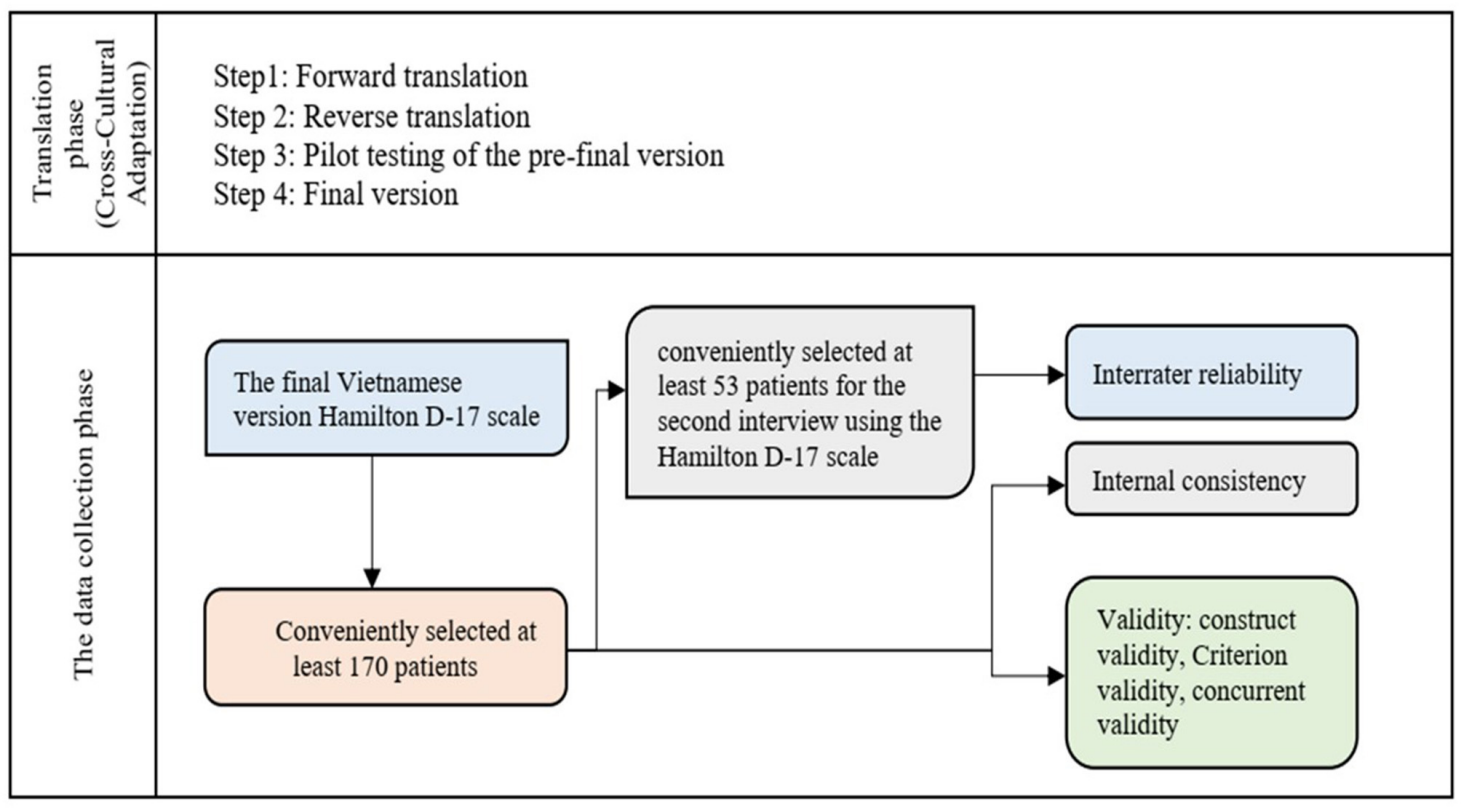
Translation of the Hamilton D-17 and study flowchart.

The Hamilton D-17 scale was translated into Vietnamese using a standard forward-backward translation approach (Figure 1). In the forward translation step, the original English version of Hamilton D-17 was translated into Vietnamese by two Vietnamese experts who were also fluent in English. These included an experienced psychiatrist and an expert in scientific research who had studied in Australia for more than 5 years. These two translators worked independently and then their translations were compared. Any differences between the two translations were discussed with the principal investigator. After reaching a consensus, a Vietnamese version of the Hamilton D-17 was finalized. Next, this Vietnamese version was translated back into English by a language specialist who was fluent in both English and Vietnamese, in which English is the mother language. In the last step, the back-translated version was compared with the original version of the Hamilton D-17 by an English native speaker who worked as an English teacher at the Western Australian International School. Since no major difference was found, the Vietnamese version of the Hamilton D-17 was used in the main study. However, to ensure the feasibility of the scale in clinical practice, the scale was sent to 8 psychiatric residents for testing on 15 patients. A discussion was organized between the researchers and the psychiatric residents to revise some minor wording of the scale.

### Study design and participants

This cross-sectional, descriptive, and validational study was conducted from January 2021–April 2021 at the Neuropsychology Clinic, University Medical Center at Ho Chi Minh City. A total of 183 patients aged ≥18 years old were recruited. Patients who had psychosis, agitation, or were unable hear, speak, or read were not invited to participate in the study.

This study was approved by Institutional Review Board (IRB) at the University of Medicine and Pharmacy at Ho Chi Minh City (approval number 708/HDDD-DHYD, dated October 12th, 2020).

### Study procedures

Patients participated in this study underwent an intensive clinical examination by a psychiatrist with 5-year experience in the field of depression. The diagnosis of depression was made by the psychiatrist based on DSM-5 and the MINI interview questionnaire. The result of this process was used as the gold standard to identify criterion validity of the Hamilton D-17. In 183 patients, 139 patients were identified as having major depressive disorders.

All 183 patients underwent a face-to-face interview by a general psychiatrist to complete a pre-defined questionnaire which contained the Hamilton D-17 and the PHQ-9. Data from these interviews were used to evaluate internal consistency, construct validity and concurrent validity. A total of 70 patients were then randomly selected to undergo a second interview with another general psychiatrist to complete the Hamilton D-17. Data from the second interview were used to evaluate inter-rater reliability.

### Measurements

Patients participated in this study underwent a face-to-face interview to complete a pre-defined questionnaire. The questionnaire contained three main parts. The first part included questions about background information such as sex, age, marital status, occupation, education and comorbidity. The second part was the Hamilton D-17 and the third part was the Patient Health Questionnaire-9 (PHQ-9). The questionnaire used for the second interview only had the Hamilton D-17. The Hamilton D-17 included 17 items: (1) depressed mood, (2) feelings of guilt, (3) suicide, (4) initial insomnia, (5) middle insomnia, (6) late insomnia, (7) work and activities, (8) retardation, (9) agitation, (10) psychiatric anxiety, (11) somatic anxiety, (12) gastrointestinal somatic symptoms; (13) general somatic symptoms, (14) genital symptoms, (15) hypochondrias, (16) weight loss, (17) insight. The PHQ-9 contained 9 items asking about symptoms of depression in the last 2 weeks. The PHQ-9 had been translated into Vietnamese and validated in previous studies.

### Data analysis

The data were analyzed by Stata 14. The internal reliability of the Hamilton D-17 scale was assessed using the Cronbach's alpha coefficient and a commonly used threshold of 0.7. Intraclass Correlation Coefficient (ICC) was used to assess inter-rater reliability, with ICC < 0.50 indicating low reliability, between 0.5 and 0.75 indicating moderate reliability, from 0.75 to 0.9 indicating good reliability and equal to or greater than 0.9 indicating excellent reliability.

The Pearson correlation coefficient was used to determine concurrent validity of the Hamilton D-17 scale based on the PHQ-9 scale. A coefficient of < 0.3 indicated a poor correlation, 0.3–0.5 demonstrated a mild correlation, and *r* > 0.5 demonstrated a strong correlation. Construct validity was evaluated through confirmatory factor analysis (CFA) based on a second-order as reported in previous studies. The model fit indices and its corresponding threshold were used as following: Chi- squared *p* ≥ 0.05, CFI ≥ 0.90, RMSEA < 0.08 with a 90% confidence interval, SRMR < 0.08, TLI ≥ 0.90. ROC analysis was conducted using the diagnosis of the experienced psychiatrists and the MINI interview as the gold standard. Area under the ROC curve (AUC) was reported with an AUC of at least 0.8 indicating good criterion validity.

## Results

### Characteristics of study participants

Among 183 participants, the mean age was 41.8 ± 14.7 years, ranging from 18 to 77 years old, (Table 1). The majority were females (71.2%), married (60%), and had at least highschool or higher (64.7%). About 15.8% of patients had at least one type of comorbidities such as hypertension, diabetes, and liver or kidney diseases. A total of 139 patients were identified as having major depressive disorders. There was no significant difference in these characteristics between patients with major depressive disorder and patients without major depressive disorder.

**Table 1:** Demographic characteristics of study participants (*N* = 183).

**Characteristics**	**All (%)**	**Major depressive period**		***p*** **-value**
		**Yes (*****N*** = **139)**	**No (*****N*** = **44)**	
		***N*** **(%)**	***N*** **(%)**	
**Gender**				
Male	52 (28.8)	40 (76.9)	12 (23.1)	0.847
Female	131 (71.2)	99 (75.6)	32 (24.4)	
**Age group (year)**				
≤ 30	49 (30.2)	42 (85.7)	7 (14.3)	0.092
31–59	112 (56.8)	79 (70.5)	33 (29.5)	
≥60	22 (12.9)	18 (81.8)	4 (18.2)	
**Mariage status**				
Single	53 (30.2)	42 (79.0)	11 (21.0)	0.768
Married/cohabitation	116 (62.6)	87 (75.0)	29 (25.0)	
Divorced/separated/widowed	14 (7.2)	10 (71.4)	4 (28.6)	
**Occupation**				
Officer/employee	47 (25.2)	35 (75.5)	12 (25.5)	0.267
Farmer/worker	28 (16.5)	23 (82.1)	5 (17.9)	
Students	14 (7.9)	11 (78.6)	3 (21.4)	
Housewife	31 (13.7)	19 (61.3)	12 (38.7)	
Other	63 (36.7)	51 (81.0)	12 (19.0)	
**Educational degree**				
Elementary school and below	25 (12.9)	18 (72.0)	7 (28.0)	0.757
Secondary school	42 (22.3)	31 (73.8)	11 (26.2)	
Highschool	44 (25.9)	36 (81.8)	8 (18.2)	
University/college and above	72 (38.8)	54 (75.0)	18 (25.0)	
**Comorbidity**				
Yes	32 (15.8)	22 (68.8)	10 (31.2)	0.294
No	151 (84.2)	117 (77.5)	34 (22.5)	

### Reliability of Vietnamese version of the Hamilton-17 scale

Most items of the Hamilton D-17 had an item-total correlation coefficient of >0.3. The Cronbach's alpha coefficient of the whole scale was at a good level (0.83), and the Cronbach's alpha coefficient when deleting an item ranged from 0.81 to 0.85 (Table 2).

**Table 2:** Internal reliability of the Vietnamese version of the Hamilton D-17 (*N* = 183).

**Item**	**Item-total correlation coefficient**	**Item-rest correlation coefficient**	**Cronbach's alpha if item deleted**
1. Depressive mood	0.74	0.69	0.81
2. Feeling guilty	0.57	0.48	0.82
3. Suicide	0.54	0.45	0.82
4. Early insomnia	0.44	0.34	0.83
5. Middle insomnia	0.53	0.44	0.82
6. Late insomnia	0.57	0.49	0.82
7. Work and activities	0.68	0.61	0.81
8. Retardation	0.34	0.24	0.83
9. Agitation	0.36	0.25	0.83
10. Psychiatric anxiety	0.58	0.50	0.82
11. Somatic anxiety	0.57	0.49	0.82
12. Gastrointestinal somatic symptoms	0.55	0.47	0.82
13. General somatic symptoms	0.73	0.67	0.81
14. Genital symptoms	0.41	0.31	0.83
15. Hypochondrias	0.56	0.47	0.82
16. Weight loss	0.58	0.49	0.82
17. Insight	0.11	−0.001	0.85
Overall			0.83

The Hamilton D-17 scale had good to excellent level of inter-rater reliability with ICC ranging from 0.81 to 0.99. However, items about “retardation” and “agitation” had low level of inter-rater reliability (Table 3).

**Table 3:** Inter-rater reliability of the Vietnamese version of the Hamilton D-17 (*N* = 70).

**Aspect**	**ICC coefficient (95% CI)**	***p*** **-value**
1. Depressive mood	0.81 (0.71–0.88)	<0.001
2. Feeling of guilty	0.97 (0.95–0.98)	<0.001
3. Suicide	0.97 (0.95–0.98)	<0.001
4. Early insomnia	0.90 (0.84–0.93)	<0.001
5. Middle insomnia	0.91 (0.85–0.94)	<0.001
6. Late insomnia	0.92 (0.88–0.95)	<0.001
7. Work and activities	0.94 (0.91–0.96)	<0.001
8. Retardation	0.59 (0.42–0.73)	<0.001
9. Agitation	0.31 (0.09–0.51)	0.004
10. Psychiatric anxiety	0.85 (0.77–0.90)	<0.001
11. Somatic anxiety	0.95 (0.91–0.97)	<0.001
12. Gastrointestinal somatic symptoms	0.99 (0.98–0.99)	<0.001
13. General somatic symptoms	0.96 (0.94–0.97)	<0.001
14. Genital symptoms	0.96 (0.94–0.98)	<0.001
15. Hypochondrias	0.95 (0.92–0.97)	<0.001
16. Weight loss	0.98 (0.97–0.99)	<0.001
17. Insight	0.81 (0.71–0.87)	<0.001
Hamilton D-17 total score	0.95 (0.93–0.97)	<0.001

### Validity of the Vietnamese version of the Hamilton D-17

**Figure 2:**
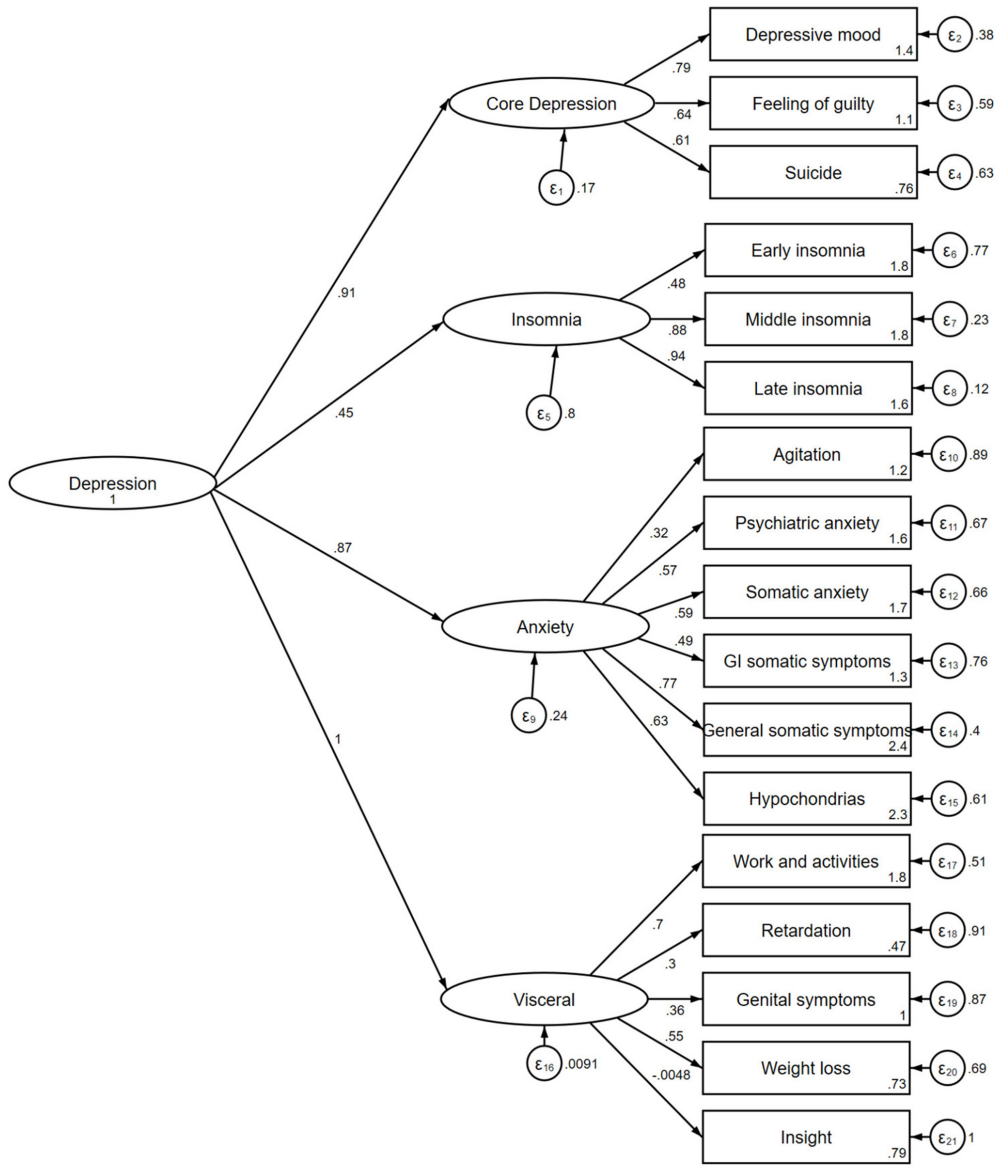
A second-order factor construct of the Vietnamese version of the Hamilton D-17.

Figure 2 presents a second-order factor construct of the Vietnamese version of the Hamilton D-17. All model fit indices indicated that the construct of the scale fitted the data well, including SRMR = 0.066, RMSEA = 0.053 (90% CI 0.036–0.069), CFI = 0.93, TLI = 0.92. Most factor loadings of four domains were at moderate to good level including core depressive (0.61–0.79), insomnia (0.48–0.94), anxiety (0.32–0.77), somatic (−0.048 to 0.70). Most factors explained the variability of variables measured, except for item about disease insight.

**Figure 3:**
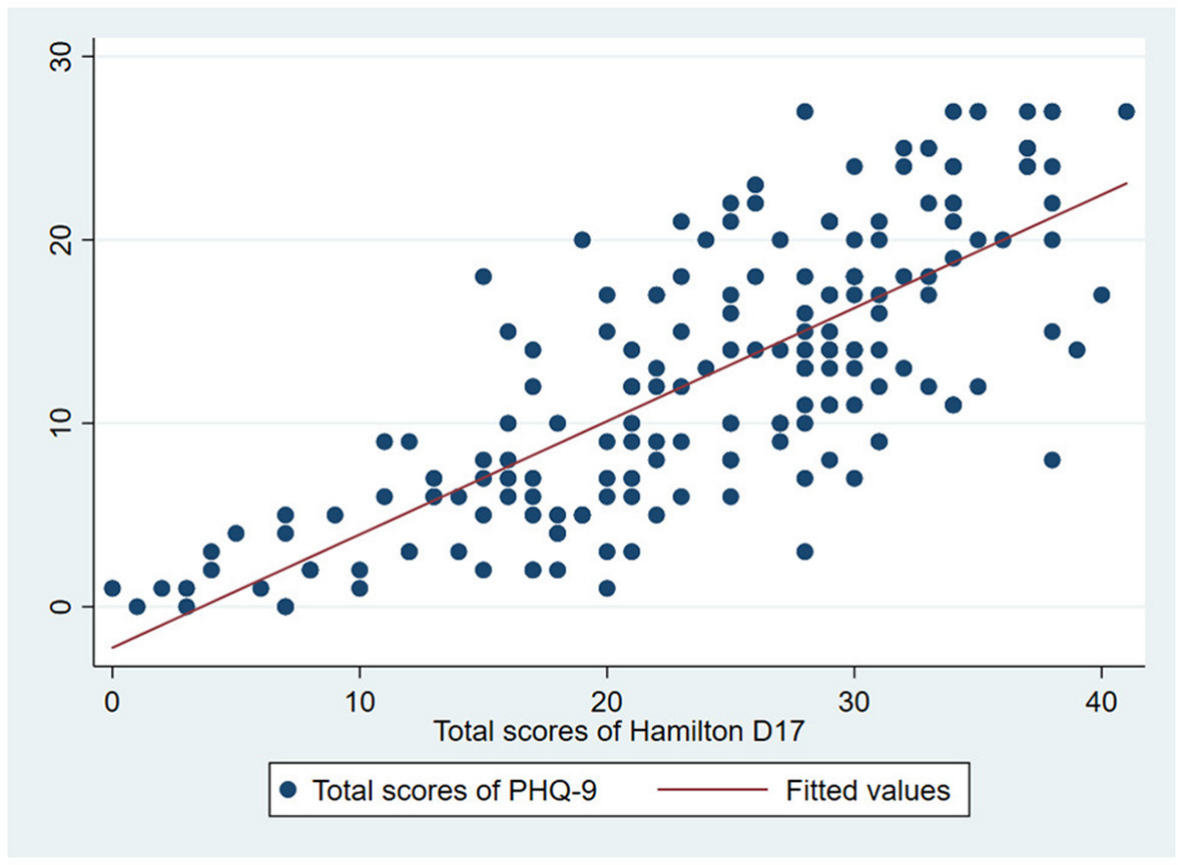
Correlation between the score of Hamilton D-17 and the score of PHQ-9.

The Hamilton D-17 and the PHQ-9 had a strong degree of correlation with correlation coefficient of r = 0.77 (*p* < 0.001) (Figure 3). This indicated a high level of concurrent validity of the Vietnamese version of the Hamilton D-17.

**Figure 4:**
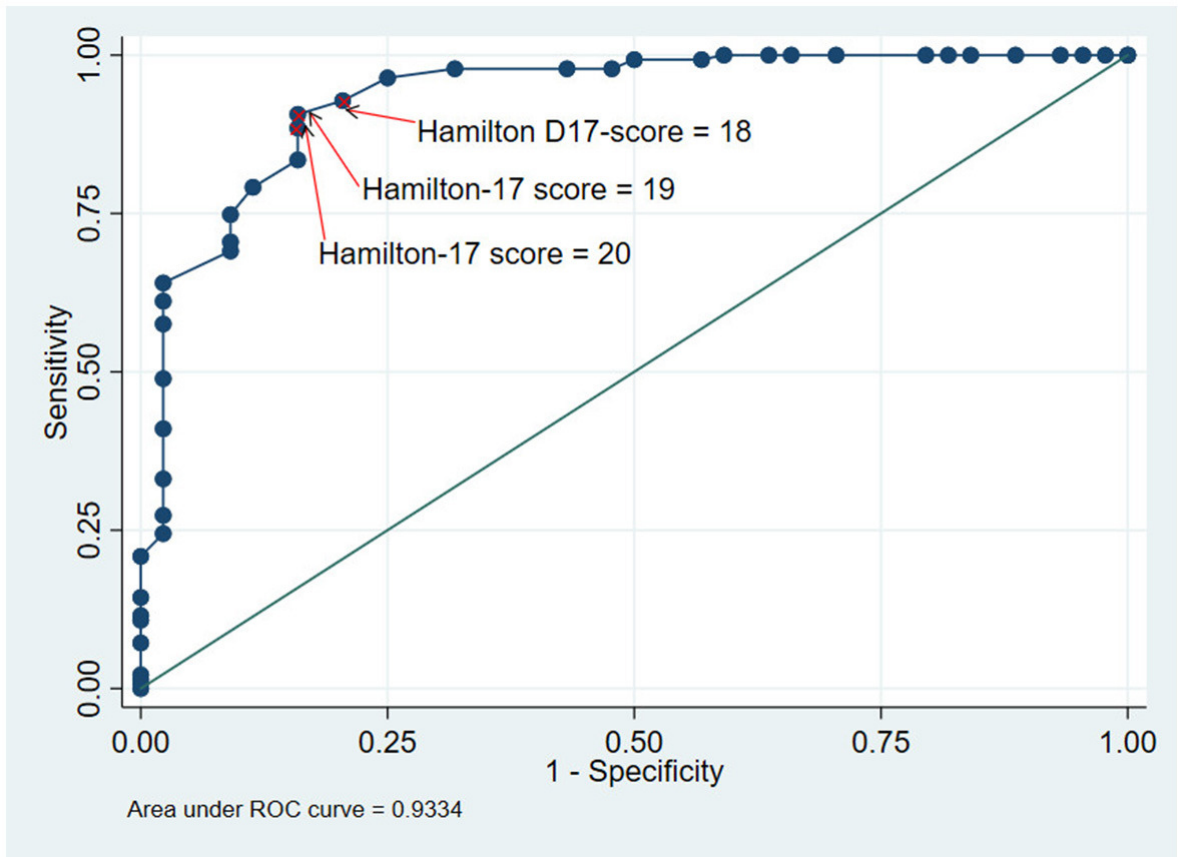
ROC curve of the Vietnamese version of the Hamilton D-17.

The Hamilton D-17 scale had excellent level of accuracy with the area under ROC curve of 0.93 (0.88–0.98) (Figure 4). This indicated criterion validity of the Hamilton D-17. Table 4 shows the predictive properties of the Hamilton D-17 at different cut-off. Although the cutoffs of 18, 19 or 20 had good discriminant ability of the scale in identify patients with depression, the cutoff of 19 was optimal. At this cutoff, sensitivity and specificity were 90.6% and 84.1%, respectively.

**Table 4:** Predictive properties of the Vietnamese version of the Hamilton D-17 scale at different cutoffs.

**Cut-off point**	**Sensitivity (%)**	**Specificity (%)**	**Positive predictive value (%)**	**Negative predictive value (%)**	**Accuracy (%)**	***d***	**Youden J index**	**Index of union**
≥15	97.8	56.8	87.7	89.3	88.0	0.43	0.55	0.41
≥16	97.8	68.2	90.7	90.9	90.7	0.32	0.66	0.30
≥17	96.4	75.0	92.4	86.8	91.3	0.25	0.71	0.21
≥18	92.8	79.5	93.5	77.8	89.6	0.22	0.72	0.14
≥19	90.6	84.1	94.7	74.0	89.1	0.18	0.74	0.12
≥20	88.5	84.1	94.6	69.8	87.4	0.19	0.73	0.14
≥21	83.5	84.1	94.3	61.7	83.6	0.30	0.68	0.19

d, distance from the cut-off point to the upper left corner.

## Discussion

The results of our study showed that the Vietnamese version of the Hamilton D-17 scale had high level of internal reliability with a high value of Cronbach's alpha coefficient. Our results are consistent with other versions of the Hamilton D-17 such as the Chinese version evaluated in 329 patients with depression (Cronbach's alpha ≥ 0.7) (13) or the Spanish version validated in 135 patients (Cronbach's alpha = 0.72) (12) and the Turkish version studied in 134 patients with depression (Cronbach's alpha = 0.75) (11). In addition, our results showed high level of inter-rater reliability. This result is similar to the Chinese version (ICC = 0.92) (13). Other versions of Hamilton D-17 in different languages such as Spanish and Turkish, also showed a strong correlation between the evaluators' results with the correlation coefficients of 0.8 or greater (11, 12).

Regarding construct validity, our study validated a four-factor second-order model as suggested by Cole et al. (15). The factor analysis confirmed that this construct on the Vietnamese version of the Hamilton D-17 fitted the data well. Thus, construct validity of the scale is confirmed in our study. In addition, the concurrent validity of the Vietnamese version of the Hamilton D-17 was checked through a correlation coefficient with the PHQ-9 score. The Hamilton D-17 and PHQ-9 scales were used to screen and measure the severity of depression. In Vietnam, the PHQ-9 scale has been assessed for its reliability and validity by Nguyen et al.'s (16) study on 2,498 lesbians and Nguyen et al.'s (17) study on 402 first-year medical students. Our study showed that the Hamilton D-17 was strongly and significantly correlated with the PHQ-9 scale, confirming the concurrent validity of the scale. This result is similar to a study by Chen et al. on 634 patients aged ≥60 years at a primary care facility (*r* = 0.66; *p* < 0.001) (18).

Although the Hamilton D-17 was not used as a diagnostic tool, the area under the ROC curve indicated that the Hamilton D-17 scale had a very high level of accuracy. This means that the Hamilton D-17 can be used to identifypatients with major depressive disorders as accurate as using theDSM-5. Our result is similar to a study by Romera on 292 patients from 36 psychiatric centers in Spain (AUC = 0.82; 95% CI = 0.76–0.87) (19) or Ballesteros' study on 113 patients (AUC = 0.93; 95% CI = 0.86–0.99) (20). In addition, our study also suggests a cut-off to distinguish patients with and without a major depressive disorder. In our study, a value of the Hamilton D-17 of 19 or greater turns out to be the optimal cutoff with high level of sensitivity and specificity. However, when the sensitivity is prioritized or the specificity is needed, the cut-off of 18 and 20 can also be used.

Although our study demonstrated good to excellent level of validity and reliability of the Hamilton D-17 scale for evaluating patients with depression at the neuropsychiatric clinic, the present study still had some limitations. First, because most study patients were from the South of Vietnam, the results may be different from region to region. Second, due to time constraints and limited resources available, the present study was conducted using the convenient sampling approach, and thus the randomness of sample selection was absent. This affects the generalizability of our study's findings. Finally, although our gold standard for diagnosis of depression was based on the DSM-5 which is used by many physicians, there might be differences in diagnosis between doctors. However, typical symptoms were unlikely to differ, and thus the likelihood of misclassification for the gold standard in our study remained minimal.

## Conclusion

The present study showed that the Vietnamese version of the Hamilton D-17 scale in has a high level of internal reliability and inter-rater reliability. The construct validity, concurrent validity and criterion validity were confirmed in our study. Due to its advantages, the Vietnamese version of the Hamilton D-17 scale should be used in clinical practice.

## Data availability statement

The raw data supporting the conclusions of this article will be made available by the authors, without undue reservation.

## Ethics statement

The studies involving human participants were reviewed and approved by Ethics Committee of Biomedical Research of University of Medicine and Pharmacy at Ho Chi Minh City. The patients/participants provided their written informed consent to participate in this study.

## Author contributions

Conceptualization and validation: PL-N-T, TN-D-U, AT-N-Q, TT, LN-T, DD-V, and SD-Q. Methodology and formal analysis: PL-N-T, TN-D-U, AT-N-Q, TT, and DD-V. Software: PL-N-T, TN-D-U, and AT-N-Q. Writing—original draft preparation: PL-N-T, TN-D-U, AT-N-Q, TT, and SD-Q. Writing—review and editing: PL-N-T, LN-T, TT, DD-V, and SD-Q. All authors contributed to the article and approved the submitted version.

## Footnotes

^1^ https://sdh.hmu.edu.vn/news/cID360_nghien-cuu-dac-diem-lam-sang-tram-cam-va-mot-so-yeu-to-lien-quan-o-benh-nhan-dai-thao-duong-typ-2.html
